# MRI in Ribbing disease—a case report

**DOI:** 10.3109/17453670903316876

**Published:** 2009-10-01

**Authors:** Michele Gaeta, Sergio Vinci, Chiara Costa, Rosaria Oliviero, Silvio Mazziotti

**Affiliations:** ^1^Department of Radiological Sciences, University of Messina, Via Consolare ValeriaMessinaItaly; ^2^Department of Social and Territory Medicine, University of Messina, Via Consolare ValeriaMessinaItaly

## Introduction

**Figure 1.**
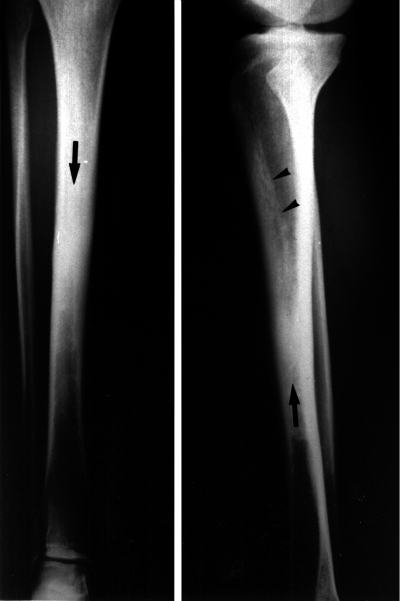
The right tibia showing endosteal sclerosis (arrows) obliterating the endosteal canal of the mid-diaphysis. A lesser degree of endosteal sclerosis can be seen in the upper diaphysis (arrowheads).

A 35-year-old woman presented in September 2008 with pain in the right tibia for 2 years. The pain had worsened in the last 3 months. As she worked as an athletic trainer in a gymnasium, a stress fracture was suspected. Radiographs of the right tibia showed cortical thickening and endosteal sclerosis in the mid-diaphysis (Figure 1). A tentative diagnosis of sclerosing osteomyelitis was made.

**Figure 2.**
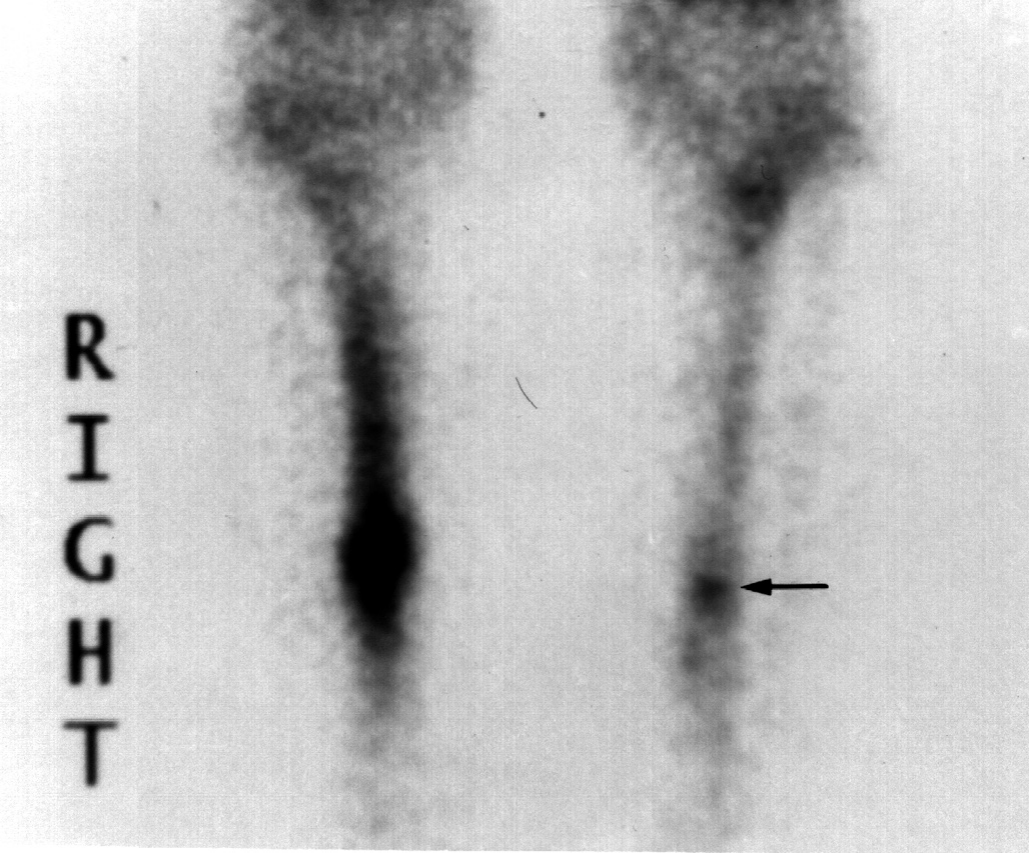
Scintigraphy showed intense uptake in the diaphysis of the right tibia. Moreover, it revealed a focus of pathological uptake in the diaphysis of the left tibia (arrow).

A ^99m^Tc-MDP bone scan demonstrated intense uptake in the right tibia and also involvement of the left tibia (Figure 2). Conventional radiographic evaluation of the left tibia was normal. The patient had no pain in the left tibia.

Biochemical evaluations showed normal erythrocyte sedimentation rate, and no serum rheumatoid factor, anti-DNA antibodies, or antinuclear antibodies. Serum alkaline phosphatase, parathyroid hormone, ionized calcium, phosphorus, and 1,25- and 25-vitamin D were normal.

**Figure 3.**
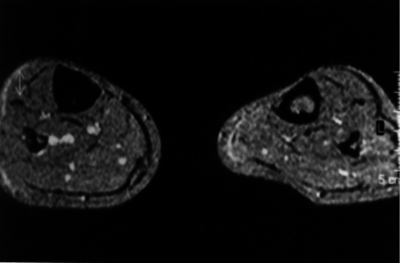
A 5-mm-thick axial T1-weighted image through the mid-tibia confirming massive endosteal sclerosis that caused obliteration of the canal of the right tibia.

**Figure 4.**
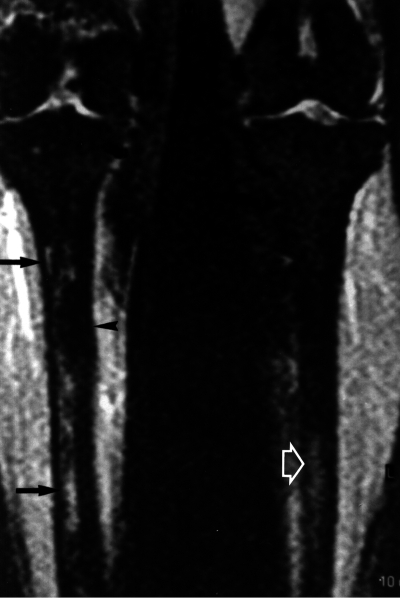
A 5-mm-thick coronal STIR image revealing endosteal sclerosis (arrowhead) with associated hyperintense edema (black arrows) in the endosteal canal of the right tibia. Moreover, slight hyperintensity in the endosteal canal of the left tibia (white arrow) can be seen.

MRI examination of both tibias confirmed the presence of sclerosis and bone marrow edema in the right tibia as well as bone marrow edema in the diaphysis of the left tibia (Figures 3 and 4). Biopsy of the right tibia showed an unspecific reactive cortical thickening with fibrosis. Attempts at culture failed to grow any organism.

On the basis of the medical history, radiographic images, and MRI images, the diagnosis of Ribbing disease was made. The patient was treated with oral non-steroidal analgesics for 2 months, with modest pain relief. 3 months after discontinuation of therapy, the pain abated further.

## Discussion

Ribbing disease is a rare condition characterized by the formation of exuberant but benign endostal and periostal new bone. It usually affects tibias and femurs. Pain is present in most patients (Ribbing 1949, Paul 1953, Seeger et al. 1996). It was first observed in 1949 by Ribbing, who described 4 siblings with asymmetric diaphyseal sclerosis in the long bones (Ribbing 1949).

Ribbing disease presents after puberty; it is either unilateral or asymmetrical and asynchronously bilateral, and has been reported only in long bones. Ribbing disease may appear to be identical to Camurati-Engelmann disease, a similar bone sclerosing dysplasia. However many clinical differences exist. Neurological abnormalities associated with Camurati-Engelmann disease are absent in Ribbing disease. Camurati-Engelmann disease presents during childhood, is bilateral and symmetric, and involves both long bones and skull (Kaftori et al. 1987, Shier et al. 1987, Seeger et al. 1996).

Radiologically, the differential diagnosis of Ribbing disease includes osteosarcoma, osteoid osteoma, osteomyelitis, and stress fracture (Kaftori et al. 1987, Shier et al. 1987, Abdul-Karim et al. 1988, Furia and Schwartz 1990, Chanchairujira et al. 2001). Until 1996, only 13 cases of Ribbing disease had been reported in the literature. In 1996, Seeger et al. illustrated imaging features on plain radiography, CT, and bone scan of 6 patients with this disease (Seeger et al. 1996).

Recently, Ziran et al. (2002) claimed the first report of MRI appearance of Ribbing disease in a 39-year-old man presenting with bilateral tibial pain and characteristic diaphyseal sclerosis of both tibias visible on conventional radiography and CT. The MRI images showed both periosteal and endosteal thickening associated with bone marrow signal abnormality consistent with marrow edema. In our case, the patient complained only of unilateral pain. Furthermore, conventional radiography showed only unilateral tibial sclerosis. MRI imaging confirmed diaphyseal sclerosis of the left tibia, but also identified the presence of bone marrow edema in both tibias—which was the only MRI abnormality visible in the left tibia.

Our findings confirm that marrow edema is present in Ribbing disease and may be responsible, at least in part, for the pain. Moreover, MRI detection of bone marrow edema allows the differentiation of Ribbing disease from intramedullary osteosclerosis, a diaphyseal dysplasia with great clinical and radiographic similarity.

Biphosphonates have been used in some cases of Ribbing and Camurati-Engelmann diseases, with discordant outcome (De Rubin et al. 1997, Inaoka et al. 2001, Ziran et al. 2002); however, the low bone turnover that seems to characterize this disease, and the prevalence of osteoblastic activity over the osteoclastic action, is no rationale for the use of osteoclast inhibitors. It is probable that the pain relief experienced by our patient after non-steroidal anti-inflammatory drug therapy should be ascribed to the natural history of the disease and not to specific success in its treatment (Ziran et al. 2002).
